# Dairy Food Intakes, Postpartum Weight Retention, and Risk of Obesity

**DOI:** 10.3390/nu15010120

**Published:** 2022-12-27

**Authors:** Mengjie Yuan, Frank B. Hu, Yanping Li, Howard J. Cabral, Sai Krupa Das, Jude T. Deeney, Lynn L. Moore

**Affiliations:** 1 Department of Medicine, Preventive Medicine and Epidemiology, School of Medicine, Boston University, Boston, MA 02118, USA; 2 Department of Nutrition, Harvard T. H. Chan School of Public Health, Boston, MA 02115, USA; 3 Channing Division of Network Medicine, Department of Medicine, Brigham and Women’s Hospital and Harvard Medical School, Boston, MA 02115, USA; 4 Department of Biostatistics, School of Public Health, Boston University, Boston, MA 02118, USA; 5 Energy Metabolism, Jean Mayer USDA Human Nutrition Center on Aging, Tufts University, Boston, MA 02111, USA; 6 Department of Medicine, Endocrinology, Diabetes, Nutrition & Weight Management, School of Medicine, Boston University, Boston, MA 02115, USA

**Keywords:** dairy foods, yogurt, cheese, postpartum weight retention, postpartum obesity, pregnancy, healthy eating

## Abstract

Excessive postpartum weight retention puts women at risk for health problems. This study aimed to investigate the effects of dairy foods on weight retention and risk of obesity in postpartum women in the Nurses’ Health Study II. Weight was reported every 2 years. We identified the pre-pregnancy and postpartum exams that were approximately 2 years before and after the birth year. Dairy consumption was averaged during these 4 years. Linear models were used to assess postpartum weight retention. Multivariable models were used to estimate risk of obesity. Women with higher yogurt (≥2 servings/week vs. <1 serving/month) intakes had 0.61 pounds less postpartum weight retention. Consuming ≥ 5 cheese servings/week was associated with 0.63 pounds less weight retention than the lowest intake. Among sedentary women, only yogurt intake was associated with lower risk of postpartum obesity (RR: 0.84; 95% CI: 0.71–1.00), though of borderline statistical significance. Among women with less healthy diets, yogurt consumption was also associated with lower postpartum obesity risk (RR: 0.70; 95% CI: 0.57–0.85). In sum, higher yogurt and cheese intakes were associated with less postpartum weight retention and among higher risk women (sedentary or lower diet quality) greater yogurt intake was associated with lower risks of postpartum obesity.

## 1. Introduction

Pregnancy represents a uniquely vulnerable time for young women for excessive weight gain and retention, thereby promoting long-term health problems [1]. In the US, the obesity prevalence for women between 20 and 39 years of age was 39.7% in 2017–2018 [2]. Data from 29 states reported that 68% of women had gestational weight gains outside of the recommendations set by the Institute of Medicine and many of these women fail to lose their excess gestational weight after childbirth [3]. A recent study revealed that 75% of women weighed more one year postpartum than they did before pregnancy, with approximately 47% of them retaining more than 10 pounds (lbs), and 24% more than 20 lbs [4]. Even for women with a normal pre-pregnancy body mass index (BMI), one-third became overweight or obese by one year postpartum [4]. Average postpartum weight retention ranges from 1.1 to 6.6 lbs. Women who are obese or overweight before pregnancy are at even greater risk of substantial postpartum weight gain [5]. Further, postpartum weight retention is a predictor of obesity later in life. In one study, women who lost their pregnancy-related weight within 6 months of delivery had gained an average of 5.3 lbs one decade later, while those who failed to lose their pregnancy weight had gained 18.3 lbs [6]. Research has linked excessive postpartum weight retention with many subsequent health problems such as hypertension, diabetes, and breast cancer [7,8,9].

Dairy foods are rich source of protein, minerals, vitamins, and other bioactive components that are vital for the optimal growth and development of the fetus. The Dietary Guidelines for Americans recommend 3 servings per day (s/d) of dairy foods for women during pregnancy and lactation [10]. Due to the high fat content of some dairy products, the impact of dairy foods on weight change has been controversial. Evidence to date from clinical trials and observational research suggests that dairy consumption does not increase body weight in long-term studies. In addition, some short-term studies have shown a greater weight-reducing effect of dairy consumption than the control group in the setting of energy-restriction [11]. Different types of dairy products may exert different effects on weight change. Data from three large cohorts of healthcare professionals including the Nurses’ Health Study I (NHS I), the Nurses’ Health Study II (NHS II), and the Health Professionals Follow-Up Study (HPFS) found that the intake of yogurt (but not milk or cheese) was strongly associated with less weight gain [12]. Results from the Framingham Offspring Study suggest that yogurt intake, compared with other types of dairy, was associated with lower annualized weight change and less incremental gain in waist circumference [13]. However, to our knowledge there is no study that has directly examined the effects of total dairy and individual dairy products on postpartum weight retention.

Therefore, the purpose of this study was to examine the independent effects of total dairy as well as individual dairy product intakes on postpartum weight retention and risk of obesity using data from women in NHS II.

## 2. Materials and Methods

Sample Characteristics

NHS II was launched in June of 1989 with the enrollment of 116,429 women, ages 25–42 years. On the first questionnaire, participants were asked to provide information on their medical history, anthropometrics, and lifestyle factors such as physical activity, smoking status, and alcohol use. Every 2 years after 1989, a follow-up questionnaire was sent to participants to update their data and to identify incident diagnoses of disease. The first food frequency questionnaire (FFQ) was sent in 1991 and subsequent FFQs were sent every 4 years. The response rate to questionnaires was approximately 85% to 90%.

In each biennial questionnaire until 2009, subjects were asked the year of any pregnancies that had occurred since the last questionnaire and whether they were currently pregnant. In the 2009 questionnaire, women were asked about the birth year, length of gestation and outcomes for all the previous pregnancies that lasted at least 20 weeks. For these analyses, we included only singleton live births of at least 20 weeks of gestation. There were 29,405 eligible singleton live births in 1991 (the first year with FFQ) or later. Further exclusions are shown in Table S1. We excluded pregnancies with missing pre-pregnant or postpartum weights (n = 7511); pregnancies occurring in women with missing dairy food intakes (n = 2069); excessive (≥6 s/d) total dairy intake (n = 214); and missing covariates (n = 188). We also excluded 470 pregnancies occurring in women with a diagnosis of prevalent or incident cancer, cardiovascular disease (CVD), or diabetes during the weight change period. Finally, we excluded 145 pregnancies in women with an extreme pre-pregnancy BMI (<16 kg/m^2^ or >40 kg/m^2^); 18 pregnancies occurring at a reported maternal age more than 50 years; and 414 in which there was an extreme postpartum weight change (the highest and lowest 1% of postpartum weight change distribution). These exclusions left a sample of 18,376 pregnancies for the analyses of postpartum weight change. In the obesity analyses, we excluded 1240 pregnancies in women who were already obese (BMI ≥ 30 kg/m^2^) at the pre-pregnancy exam, leaving 17,136 pregnancies for the analysis of incident obesity.

The study protocol was approved by the Institutional Review Boards of the Brigham and Women’s Hospital and the Harvard T.H. Chan School of Public Health. All analyses were approved by the Institutional Review Board of Boston University School of Medicine.

Dietary Assessment

The 131-item FFQs were collected every 4 years and requested information on how often the participant consumed each food on a list in the past year. The nine categories of consumption frequency included never or less than once per month, 1–3 servings per month (s/m), 1 serving per week (s/w), 2–4 s/w, 5–6 s/w, 1 s/d, 2–3 s/d, 4–5 s/d, and ≥6 s/d. The following foods were included in the total dairy analysis: milk (whole milk, 1 or 2% fat milk, and skim milk), yogurt, and cheese (cottage, ricotta, and other cheese). Butter, cream, and cream cheese were excluded due to low calcium content. A standard serving size was 1 cup for milk and yogurt, 1 slice or 1 ounce serving for other cheese (e.g., American, cheddar), and 0.5 cup for cottage or ricotta cheese. Nutrient intakes were calculated based on consumption of all the foods reported in the FFQ. Alternative Healthy Eating Index (AHEI) scores were calculated for individual participants based on their food and nutrient intakes from each FFQ [14]. Dairy products were not a component of the AHEI score.

Assessment of Weight and BMI

Height and weight were self-reported at enrollment and weights were updated on all biennial follow-up questionnaires. BMI was calculated for each questionnaire cycle by using weight divided by baseline height, squared. Overweight was defined as BMI ≥ 25 kg/m^2^, and obesity was defined as BMI ≥ 30 kg/m^2^.

Assessment of Other Factors

Age at childbirth was calculated by subtracting the mother’s birth year from the year of childbirth. Birth order (first, second, third, etc.) for each live born delivery was self-reported on the 2009 questionnaire. Physical activity was assessed on each biennial questionnaire by asking participants about the average time per week spent on specific activities such as jogging, running, biking, playing tennis, swimming, etc. We summed the hours per week for each activity to derive total activity hours per week

Statistical Analysis

**Figure 1:**
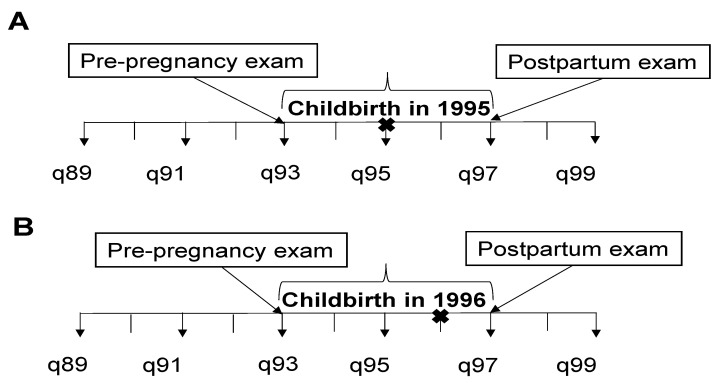
Study Timelines for Weight Retention Analyses. (**A**) represents the situation when childbirth happened in a year with questionnaire. (**B**) represents the situation when childbirth happened in a year without questionnaire. Q stands for questionnaire, and X indicates the timepoint of childbirth.

For the analyses of postpartum weight retention, we began by selecting a 4-year period surrounding childbirth for the assessment of dietary intake and weight change as shown in Figure 1. Since questionnaires were sent every 2 years and we did not have an exact date of birth, the pre-pregnancy and postpartum exams were defined according to whether the birth occurred during a questionnaire year or not. As shown in the diagram, when childbirth happened in a year with a questionnaire, we used pre-pregnancy information from the questionnaire 2 years earlier and postpartum information from 2 years after the birth. If the birth occurred in between questionnaire years, we took pre-pregnancy information from two exams prior to the birth year (since the woman may have been pregnant already at the exam just prior to childbirth), and postpartum information from the exam after childbirth. In this way, weight change from pre-pregnancy to the postpartum visit was taken from a 4-year period for each woman. Postpartum weight retention as calculated as postpartum weight minus pre-pregnancy weight.

To assess the association between the dairy consumption and postpartum weight retention, we used mean dairy intakes from the 4-year period surrounding the delivery as the exposure. Sensitivity analyses and the intake distribution for each type of dairy were used to categorize intakes as follows: total dairy (<1 s/d, 1 s/d–<2 s/d, and ≥2 s/d), yogurt (<1 s/m, 1 s/m–<2 s/w, and ≥2 s/w), milk (<1 s/d, 1 s/d–<2 s/d, and ≥2 s/d), and cheese (<2 s/w, 2 s/w–<5 s/w, and ≥5 s/w). The outcome was weight retention as a continuous variable. Multivariable general linear models were used to estimate adjusted mean postpartum weight retention in each category of intake. An exchangeable correlation matrix was used to account for within-subject correlation since women were allowed to contribute data from more than one pregnancy.

We tested a range of potential covariates including age at childbirth, birth order, breast-feeding, physical activity, AHEI score, red and processed meat, sugar sweetened beverage (SSB) intakes, energy intake, alcohol, fiber, fruits and vegetables, trans fatty acid intakes, and smoking status. Finally, we decided to retain the covariates that changed the effect estimates by more than 5% and we also adjusted each type of dairy food by other dairy foods (e.g., milk intake model was adjusted for intakes of yogurt and cheese). The covariates retained in the final model were age at childbirth (continuous), birth order (first, second, or third or later childbirth), trans fat intakes (continuous), fruits and vegetables (continuous), alcohol (continuous), and SSBs (continuous); intakes of milk, yogurt or cheese were adjusted for each other. Tests for linear trend across increasing categories of individual dairy food intake were performed by assigning the median value of the respective categories of exposure and entering this as a continuous variable into the models.

There were 17,136 non-obese women who were followed up from the pre-pregnancy exam to the postpartum exam for the occurrence of incident obesity. Multivariable Poisson regression models were used to estimate the relative risks and 95% confidence intervals for postpartum obesity associated with dairy food intakes. The same potential confounding factors were included in the final models as described in the weight change analyses. We also explored potential effect modification of dairy foods by physical activity, and diet quality as estimated by the AHEI. We selected activity and AHEI as potential effect modifiers because these healthy lifestyle factors are important strategies for weight management and they are also associated with yogurt consumption [15]. To test potential effect modification of dairy foods by these factors, we created combined categories of each dairy product with each physical activity and AHEI score. Physical activity was dichotomized as higher (≥2 h/week, h/w) vs. lower (<2 h/w) based on the distribution of physical activity. AHEI was likewise dichotomized as higher (top 3 quintiles) vs. lower (bottom 2 quintiles) and dairy food intakes were dichotomized as follows: total dairy (<2 s/d vs. ≥2 s/d), yogurt (<1 s/m vs. ≥1 s/m), milk (<1 s/d vs. ≥1 s/d), and cheese (<2 s/w vs. ≥2 s/w). Subjects were then cross-classified into four groups based on these dichotomized groups. For example, the combined categories of total dairy and physical activity were: (1) lower physical activity (<2 h/w) + lower total dairy intake (<2 s/d), (2) lower physical activity (<2 h/w) + higher total dairy intake (≥2 s/d), (3) higher physical activity (≥2 h/w) + lower total dairy intake (<2 s/d), and (4) higher physical activity (≥2 h/w) + higher total dairy intake (≥2 s/d). Those in the lower categories for both dichotomous variables served as the referent group for each analysis.

Statistical significance was set at a two-tailed *p* < 0.05. SAS version 9.4 (SAS Institute) was used to analyses the data.

## 3. Results

The characteristics by total dairy consumption can be found in Table S2. The characteristics of subjects in the postpartum weight retention analysis are presented in Table 1. Women with higher yogurt intake were slightly older when giving birth, and tended to be more active, have a healthier overall diet, and be non-smokers. It was also more likely for higher yogurt consumers to be giving birth for the first time. In terms of nutrients intakes, women of higher yogurt group consumed more calories and more energy-adjusted intakes of protein, carbohydrates, and alcohol but less fat. There was a positive association between yogurt intake with fruits and vegetables. The consumption of SSB and red & processed meat decreased with yogurt intake. The characteristics by total dairy consumption can be found in Table S2.

Table 2 demonstrates postpartum weight retention by intake of different dairy foods. After adjusting for potential confounders, women who consumed more than 2 s/w of yogurt had 0.61 lbs (p-trend = 0.031) less weight retention then women who consumed less than 1 s/m. Higher cheese (≥5 s/week vs. <2 s/week) intake was linked with 0.63 lbs (p-trend = 0.003) less weight retention. When we added pre-pregnancy BMI to the multivariable model, the favorable effects of yogurt and cheese on weight retention remained and were only very slightly attenuated (p-trend = 0.035 for yogurt intake; p-trend = 0.013 for cheese intake). There was no clear effect of total dairy or milk intake on postpartum weight retention in any model.

We explored the effects of dairy food intakes on postpartum obesity risk in Table 3. Moderate and higher yogurt intakes were associated lower risks of obesity (i.e., a statistically significant 20% (95% CI: 0.69–0.93) lower risk for moderate intake and a non-statistically significant 15% (95% CI: 0.70–1.03) lower risk for higher intake). There were no associations between total dairy, milk, or cheese intakes and obesity risk. Adding pre-pregnancy BMI attenuated any favorable effects of yogurt on obesity.

Table 4 shows the association of different dairy food intakes in combination with activity and AHEI scores. Higher activity alone was generally associated with lower risks of obesity. Higher yogurt intake among those with lower activity levels reduced the risk of obesity by 16% (95% CI: 0.71–1.00). Higher yogurt and activity together led to a statistically significant 39% (95% CI: 0.50–0.74) lower risk of postpartum obesity compared with lower yogurt intake and lower activity. Women with more physical activity and higher cheese consumption showed a statistically significant 26% reduction in obesity risk compared with those who are more sedentary and eat less cheese. There were no statistically significant reductions in postpartum obesity risk observed for total dairy or milk intake alone. Among women with lower diet quality (AHEI) scores, higher yogurt intake was associated with 30% (95% CI: 0.57–0.85) lower risk of postpartum obesity. However, higher AHEI scores alone were associated with statistically significant reductions in risk of postpartum obesity.

## 4. Discussion

The current study finds that higher intakes of yogurt and cheese were associated with less postpartum weight retention. Further, yogurt intake was associated with a 16% lower postpartum obesity risk in more sedentary women, and a 30% lower risk in women with lower diet quality scores. Higher activity levels and higher AHEI scores were both independently associated with less postpartum obesity risk. Further, both yogurt and cheese intakes had beneficial effects on postpartum obesity risk when combined with higher activity levels. There was no clear association between total dairy or milk consumption and postpartum weight gain or development of obesity. To our knowledge, this was the first study that focused on the association between dairy food intakes and postpartum weight retention. Our study provides important evidence that total dairy or individual dairy foods do not lead to increased postpartum weight retention or obesity risk. In contrast, incorporating cheese or yogurt in particular into the maternal diet could have favorable effects on attenuating postpartum weight retention and improving the health of women at this critical stage.

Dairy foods are an excellent source of essential nutrients for fetal growth and studies found that maternal consumption of dairy foods has positive effects on fetal femur growth, and infant birthweight [16]. The Dietary Guidelines recommend the consumption of dairy products as an important part of a healthy and balanced diet for women during pregnancy and early postnatal life [10]. It is possible that at least part of the beneficial effects of dairy foods on the offspring of pregnant women are a result of intermediate effects on maternal body weight. We were unable to find research that investigated the potential impact of dairy foods on postpartum weight change directly. However, a few studies have reported the association between dairy intake and gestational weight retention. In one prospective analysis of pregnant Portuguese women, Abreu et al. measured the dietary intake of milk, cheese, and yogurt in pregnant women during both the first and second trimesters. After adjusting for potential confounding, change of total dairy intakes from the first to the second trimesters had a significant inverse association with gestational weight gain. Moreover, the increase in consumption of individual dairy products including milk, yogurt and cheese were all inversely associated with gestational weight gain, although these associations were not statistically significant [17]. However, in a study of 1388 women from the Project Viva cohort, the authors reported that total dairy consumption during pregnancy was associated with an increased risk of excessive gestational weight gain (OR: 1.08; 95% CI: 1.00–1.17, per s/d) [18]. They also found that this association did not appear to be driven by whole fat dairy foods, because both low fat (OR: 1.08; 95% CI: 0.98−1.18, per s/d) and whole fat (OR: 1.06; 95% CI: 0.94−1.20, per s/d) dairy foods were similarly associated with increased risk of excessive gestational weight gain. Gestational weight gain is a strong predictor of postpartum weight retention. A meta-analysis of 9 observational studies found that women with excessive gestational weight gain retained significantly more weight postpartum [19].

Research in adult populations has supported favorable effects of yogurt consumption on weight management. Higher yogurt intake was reported to be linked with lower BMI, lower body weight gain, smaller waist circumference (WC) and lower body fat in the general adult population [20]. In a previous study of participants from NHS I, NHS II and HPFS, 1 serving of yogurt per day was associated with 0.82 lbs less weight gain over 4 years after adjusting for other lifestyle and dietary variables [12]. Notably, yogurt intake was also reported to have significant effects on attenuating central adiposity, an important component of metabolic syndrome. In a large prospective study from Spain, adults with higher yogurt consumption had a 15% lower risk of developing central adiposity over 6 years [21]. Results from the Framingham Offspring Study also found that participants who consumed more yogurt (≥3 s/w vs. <1 s/w) had a 0.13 cm lower annualized increase in WC (p-trend = 0.008) [13]. Evidence suggests that childbearing preferentially contributes to the increase of visceral fat [22]. Visceral fat is metabolically active and contributes to a variety of adverse health outcomes such as insulin resistance, diabetes, and CVD [23]. Given the increased visceral fat in postpartum women, the weight-reducing effects of yogurt could provide important benefits for women at this critical stage.

Although cheese has long been perceived to promote weight gain due to its high fat content, most studies so far have suggested that it either has no effect or a protective effect on weight gain. A previous study of three large cohorts of healthcare professionals (NHS I & II and HPFS) found a null association of cheese intake and weight change. Similarly, in the Framingham Offspring Study, higher cheese intake (≥3 s/w vs. <1 s/w) did not lead to any difference in weight change or WC in 3440 participants [13]. In contrast, Rosell et al. found that women who regularly consumed more than 1 s/d of cheese had a 30% (95% CI: 0.59–0.84) lower risk of weight gain compared with those who consumed less than 1 s/d [24]. The variation in nutrient profile among different types of cheese may have different effect on weight management. A cross-sectional study examined the association between weight and 3 subgroups of cheese including fresh, mature and processed cheese in 1081 Basque adults. The study found both fresh and processed cheese were inversely associated with excess weight, while no association was observed for mature cheese [25]. However, few studies are available on the effects of different cheeses on weight management. FFQs in NHS2 provided limited information for us to stratify the cheese by type. Future studies are needed to examine this question.

The mechanisms by which yogurt or cheese may benefit weight change are not clear, but several explanations have been proposed. The bacteria present in fermented dairy products have anti-inflammatory properties. Some evidence indicates that fermented dairy foods have stronger effects on lowering chronic inflammation than non-fermented dairy foods, and these findings could reduce the risk of obesity [26]. Additionally, the products derived from the fermentation of milk such as bioactive peptides and probiotics have been reported to play a role in weight management. Bioactive peptides have been shown to be effective in preventing obesity by suppressing appetite, regulating adipocyte differentiation, and impacting the activity of lipase [27,28]. Probiotics are live bacteria that boost human health through the digestive system. It has been postulated that probiotics may favorably modulate gut microbiota and prevent against obesity [29,30]. A meta-analysis of 15 randomized controlled clinical trials showed that probiotic administration (for 3 to 12 weeks) significantly reduced weight by 0.60 kg compared with the control group. It is also reported that longer-term interventions and single species of probiotics have stronger weight reducing effects [31]. While there here are numerous types of probiotics that may play different roles in regulating weight, Lactobacillus and Bifidobacterium are two major genera that have long been used in the fermentation of yogurt. Certain strains of Lactobacillus have been shown to exhibit benefits on weight loss. A clinical trial with 210 healthy Japanese healthy adults found that consumption of Lactobacillus gasseri SBT2055 resulted in an 8.5% reduction of abdominal visceral fat, and cessation of consumption led to re-gaining of abdominal fat [32]. Another clinical trial reported that intake of yogurt with Lactobacillus amylovorus or Lactobacillus fermentum conferred significant fat loss in healthy but overweight persons [33]. In individuals with abdominal adiposity, intake of Bifidobacterium animalis subsp. lactis CECT 8145 significantly decreased waist circumference and BMI [34]. However, some studies suggest that other strains of Lactobacillus and Bifidobacterium may not promote weight or fat loss. Yin et al. compared the effects of 4 different Bifidobacterium strains on body weight in an obese rat model and found one strain reduced body weight, one strain increased body weight, while the other two had no effects [35]. Another 2016 systematic review revealed the effects of probiotics on body weight differed by species and strain. The study found that some Lactobacillus strains even promoted weight gain [36], suggesting that the association between probiotics and weight is not homogenous. Unfortunately, NHS2 did not gather information yogurt brands or the probiotic content of yogurt.

Increased calcium has also been found to inhibit lipogenesis, stimulate lipolysis, promote lipid oxidation, and increase the excretion of fecal fat [29,30]. Although a cup of yogurt has a similar amount of calcium as one cup of milk, yogurt is believed to improve the bioavailability of calcium due to its acidity [37]. Thus, the improved bioavailability may account for the specific weight-reducing effects of yogurt. Further, despite the fact that cheese has been perceived as a food that promotes weight gain due to its high content of fat, recent studies suggested that certain dairy fatty acids such as conjugated linoleic acid, butyric acid, and palmitoleic acid have positive effects on reducing adiposity [38].

We also explored the effects of dairy food consumption combined with physical activity and diet quality on postpartum obesity risk. Because of the need to feed and nurse infants, women are thought to be more sedentary during pregnancy and the early postnatal years. Increased physical activity might be an effective strategy to reduce postpartum weight retention. However, a previous clinical trial showed that 12 weeks of a moderate aerobic exercise intervention did not promote weight loss in lactating women [39]. In addition, Lovelady et al. found that a 10-week program combining moderate physical activity and calorie restriction was associated with weight loss in women during the postpartum period [40]. In the current results, we found that the women with higher physical activity alone had reduced risk of obesity. The physical activity in the current study represents long-term activity instead of a short-term intervention as in the previous clinical trial. This could explain why physical activity had obesity-reducing effects in the current study but not in some earlier studies. Interestingly, the current analysis also found that the combination of activity with yogurt or cheese intake resulted in an even lower postpartum obesity risk than activity alone. These results together suggested that adequate exercise combined with yogurt or cheese intakes may be effective strategies for women to combat postpartum obesity risk. Boghossian et al. investigated the association between the AHEI score and weight change in 1136 lactating women, and they found women with higher AHEI scores tended to have lower postpartum weight retention [41]. In this analysis, we observed that a higher AHEI itself contributed to decreased risk of obesity. Considering that it is much easier and more practical to change the consumption of a single food than changing the overall dietary pattern, our finding provides an effective strategy for women with a less than optimal holistic diet to manage their obesity risk after pregnancy.

There are several strengths of the present study. First, it was the first study that illustrated the effects of different dairy foods on weight in the postpartum period, a critical stage during which women are prone to the development of obesity. Secondly, the current analysis was based on the large NHS II cohort, so we had a larger sample size than most previous studies that focused on postpartum women. However, the findings of our study should also be assessed in light of the limitations of the data. First, we do not have the specific month and day of birth, which prevented us from calculating the exact postpartum time period. Secondly, the dietary data were collected using an FFQ, which may be subject to measurement error. However, a previous study has confirmed the reasonable accuracy of estimation of dairy consumption using the FFQ compared with a 7-day dietary record [42]. The correlation coefficients for skim milk, whole milk, yogurt, cottage cheese and hard cheese were 0.81, 0.62, 0.94, 0.80, and 0.57, respectively. Thirdly, the weights were self-reported in our study. Although the previous study found a high correlation (r = 0.96) between self-reported weights and measured weights, reported weights were relatively lower than the measured weights [43], which could lead to some underestimation of weight retention, if self-reported postpartum weights were subject to greater underestimation than pre-pregnancy weights [43]. Finally, for under-reporting of postpartum weight to bias the estimated effects in this study, the report of postpartum weight would need to be differentially associated with dairy intake categories. This seems unlikely since postpartum weight was reported approximately two years after delivery. Thus, the weight change between those two weights may be less biased than either reported weight alone.

## 5. Conclusions

In conclusion, our results suggest that higher consumption of yogurt and cheese are associated with less postpartum weight retention. Women with higher physical activity levels and higher intakes of yogurt had a 39% lower risk of incident postpartum obesity. The AHEI alone was also associated with a lower obesity risk. Incorporating yogurt or cheese intake into the diet may be an effective strategy for women to manage their weight during the postpartum period.

## Figures and Tables

**Table 1:** Diet and Lifestyle Characteristics of Women in the Postpartum Weight Retention Analysis by Categories of Yogurt Intake throughout the 4 Years Surrounding Pregnancy ^1^.

	Yogurt Intake		
	1 s/m	1 s/m–<2 s/w	≥2 s/w
N of live births	5744	8871	3761
	Mean (SD)		
Age at birth (years)	34.7 (3.8)	35.2 (3.8)	35.6 (3.9)
Pre-pregnant BMI (kg/m^2^)	23.3 (4.0)	23.2 (3.8)	23.0 (3.7)
Activity (h/w)	2.0 (2.5)	2.4 (2.7)	3.1 (2.9)
AHEI score	44.2 (10.3)	47.8 (10.0)	51.3 (10.2)
Energy intake (kcals/day)	1826 (540)	1903 (516)	2046 (514)
Energy-adj protein (g/day)	83.6 (14.0)	85.0 (12.7)	86.5 (12.9)
Energy-adj carbohydrate (g/day)	226.8 (33.8)	232.2 (29.9)	242.0 (29.0)
Energy-adj total fat (g/day)	63.7 (11.4)	60.7 (10.2)	55.9 (10.0)
Energy-adj trans-fat (g/day)	3.3 (1.2)	3.0 (1.0)	2.6 (0.9)
Energy-adj alcohol (g/day)	2.3 (5.2)	2.8 (5.0)	3.1 (5.5)
Total dairy (s/day)	1.8 (1.3)	2.1 (1.2)	2.7 (1.2)
Yogurt (s/week)	0.0 (0.0)	0.8 (0.5)	4.1 (1.9)
Milk (s/day)	1.3 (1.1)	1.4 (1.1)	1.5 (1.1)
Cheese (s/day)	0.5 (0.5)	0.6 (0.4)	0.6 (0.5)
Red and processed meat (s/day)	1.0 (0.6)	0.9 (0.6)	0.8 (0.5)
Fruits and vegetables (s/day)	3.7 (2.1)	4.4 (2.1)	5.3 (2.4)
SSB (bottles/day)	1.4 (1.4)	1.3 (1.2)	1.2 (1.2)
Pre-pregnancy smoker (%)	9.9	7.0	6.5
First birth (%)	23.7	25.7	32.1

Abbreviations: AHEI, alternative healthy eating index 2010; BMI, body mass index; Energy-adj, energy adjusted; SD, standard deviation; SSB, sugar sweetened beverages; s/d, servings per day; s/m, servings per month; s/w, servings per week. ^1^ All the dietary variables and activities variables are cross-sectional; only BMI and smoking status are pre-pregnant.

**Table 2:** Postpartum Weight Retention by Intakes of Different Dairy Foods.

		Multivariable ^1^	Multivariable + Pre-Pregnant BMI
	N	Mean of Postpartum Weight Retention (lb)	
Total dairy			
<1 s/d	3572	6.52	6.55
1 s/d–<2 s/d	6198	6.35	6.33
≥2 s/d	8606	6.30	6.28
p-trend:		0.380	0.289
Yogurt			
<1 s/m	5744	6.67	6.65
1 s/m–<2 s/w	8871	6.29	6.28
≥2 s/w	3761	6.06	6.06
p-trend:		0.031	0.035
Milk			
<1 s/d	7683	6.43	6.44
1 s/d–<2 s/d	4769	6.22	6.21
≥2 s/d	5924	6.38	6.34
p-trend:		0.923	0.706
Cheese			
<2 s/w	5493	6.66	6.62
2 s/w–<5 s/w	7519	6.37	6.32
≥5 s/w	5364	6.03	6.11
p-trend:		0.003	0.013

Abbreviations: BMI, body mass index; lb, pound; Ref, reference; s/d, servings per day; s/m, servings per month; s/w, servings per week. ^1^ Adjusted for the age at childbirth, the order of childbirth, trans fat, fruit and vegetable, alcohol, sugar sweetened beverages (Yogurt, cheese, and milk were adjusted for each other).

**Table 3:** Relative Risk of Postpartum Obesity According to Different Dairy Foods Intakes.

	N	Obesity Cases	RR (95% CI)	RR (95% CI)
			Multivariable ^1^	Multivariable + Pre-Pregnant BMI
Total dairy				
<1 s/d	3305	163	1.00 (Ref)	1.00 (Ref)
1 s/d–<2 s/d	5788	309	1.13 (0.94–1.36)	1.06 (0.90–1.25)
≥2 s/d	8043	383	1.07 (0.89–1.29)	1.08 (0.92–1.27)
Yogurt				
<1 s/m	5307	317	1.00 (Ref)	1.00 (Ref)
1 s/m–<2 s/w	8295	374	0.80 (0.69–0.93)	0.90 (0.79–1.01)
≥2 s/w	3534	164	0.85 (0.70–1.03)	0.93 (0.79–1.10)
Milk				
<1 s/d	7137	363	1.00 (Ref)	1.00 (Ref)
1 s/d–<2 s/d	4459	230	1.06 (0.90–1.25)	1.06 (0.92–1.22)
≥2 s/d	5540	262	1.02 (0.86–1.20)	1.03 (0.90–1.18)
Cheese				
<2 s/w	5124	256	1.00 (Ref)	1.00 (Ref)
2 s/w–<5 s/w	6980	363	1.04 (0.89–1.22)	1.05 (0.92–1.20)
≥5 s/w	5032	236	0.92 (0.77–1.10)	0.96 (0.82–1.12)

Abbreviations: BMI, body mass index; CI, confidence interval; lb, pound; Ref, reference; RR, relative risk; s/d, servings per day; s/m, servings per month; s/w, servings per week. ^1^ Adjusted for the age at childbirth, the order of childbirth, trans fat, fruit and vegetable, alcohol, sugar sweetened beverages (Yogurt, cheese, and milk were adjusted for each other).

**Table 4:** Risk of Postpartum Obesity According to Intake of Different Dairy Foods, Stratifying by Physical Activity and AHEI ^1^.

	Lower Activity ^2^	Higher Activity	Lower Activity	Higher Activity
	Number of Subjects		RR (95% CI)	
Dairy < 2 s/d	5215	3878	1.00 (Ref)	0.70 (0.58–0.85)
Dairy ≥ 2 s/d	4468	3575	0.95 (0.81–1.13)	0.74 (0.60–0.91)
Yogurt < 1 s/m	3406	1901	1.00 (Ref)	0.78 (0.62–0.99)
Yogurt ≥ 1 s/m	6277	5552	0.84 (0.71–1.00)	0.61 (0.50–0.74)
Milk < 1 s/d	4021	3116	1.00 (Ref)	0.72 (0.58–0.89)
Milk ≥ 1 s/d	5662	4337	1.02 (0.86–1.21)	0.76 (0.62–0.93)
Cheese < 2 s/w	2890	2234	1.00 (Ref)	0.82 (0.64–1.05)
Cheese ≥ 2 s/w	6793	5220	1.05 (0.87–1.26)	0.74 (0.59–0.91)
	Lower AHEI ^3^	Higher AHEI	Lower AHEI	Higher AHEI
	N of subjects		RR (95% CI)	
Dairy < 2 s/d	3287	5806	1.00 (Ref)	0.63 (0.51–0.77)
Dairy ≥ 2 s/d	3567	4476	0.82 (0.67–1.00)	0.68 (0.54–0.85)
Yogurt < 1 s/m	2831	2476	1.00 (Ref)	0.58 (0.45–0.74)
Yogurt ≥ 1 s/m	4023	7806	0.70 (0.57–0.85)	0.58 (0.47–0.72)
Milk < 1 s/d	2539	4598	1.00 (Ref)	0.58 (0.46–0.73)
Milk ≥ 1 s/d	4315	5684	0.84 (0.68–1.02)	0.69 (0.55–0.86)
Cheese < 2 s/w	1795	3328	1.00 (Ref)	0.70 (0.53–0.90)
Cheese ≥ 2 s/w	5059	6954	0.95 (0.77–1.19)	0.69 (0.54–0.88)

Abbreviations: AHEI, alternative healthy eating index 2010; CI, confidence interval; RR, relative risk; s/d, servings per day; s/m, servings per month; s/w, servings per week. ^1^ Adjusted for the age at childbirth, the order of childbirth, trans fat, fruit and vegetable, alcohol, sugar sweetened beverages (Yogurt, cheese, and milk were adjusted for each other). ^2^ Lower vs higher physical activity is defined as <2 vs. ≥2 h of activity per week. ^3^ Lower vs higher AHEI is defined as the lower two quintiles vs the upper three quintiles of AHEI score.

## Data Availability

Data described in the manuscript, code book, and analytic code will be made available upon request pending application and approval. Further information including the procedures to obtain and access data is described at https://www.nurseshealthstudy.org/researchers (accessed on 8 July 2022) (contact email: nhsaccess@channing.harvard.edu).

## Supplementary Materials

The following supporting information can be downloaded at: https://www.mdpi.com/article/10.3390/nu15010120/s1, Table S1: Exclusion Criteria for Weight Change Analysis; Table S2: Diet and Lifestyle Characteristics of Women in the Postpartum Weight Retention Analysis by Categories of Total Dairy Intake throughout the 4 Years Surrounding Pregnancy.
