# Supplementary material for: Dairy Food Intakes, Postpartum Weight Retention, and Risk of Obesity
Source: Nutrients. 2022 Dec 27;15(1):120. doi: 10.3390/nu15010120 (PMC9824318; doi:10.3390/nu15010120)
Supplement: Supplementary file 1 [file nutrients-15-00120-s001.zip › Supplementary.pdf]

**Table S1. Exclusion Criteria for Weight Change Analysis.**

| Criteria | Exclusions | Remaining N |
|---|---|---|
| **Start with 29,405 singleton livebirth at/after 1991:** | | 29,405 |
| Missing pre-pregnant or postpartum weights | 7,511 | 21,894 |
| Missing cross-sectional dairy food intakes | 2,069 | 19,825 |
| Total dairy intakes ≥ 6 s/d | 214 | 19,611 |
| Missing height | 17 | 19,594 |
| Missing activity | 150 | 19,444 |
| Missing smoking | 21 | 19,423 |
| Prevalent or incident cancer during follow-up | 260 | 19,163 |
| Prevalent or incident CVD during follow-up | 94 | 19,069 |
| Prevalent or incident diabetes during follow-up | 116 | 18,953 |
| Baseline BMI < 16 $kg/m^2$ | 5 | 18,948 |
| Baseline BMI > 40 $kg/m^2$ | 140 | 18,808 |
| Age at childbirth > 50 years | 18 | 18,790 |
| Postpartum weight gains > top 1%, i.e., 45 lb | 208 | 18,582 |
| Postpartum weight loss < bottom 1%, i.e., 20 lb | 206 | 18,376 |
| **Final sample size for postpartum weight change analyses** | | **18,376** |

*We further excluded 1240 prevalent obese subjects for obesity analysis.

**Table S2. Diet and Lifestyle Characteristics of Women in the Postpartum Weight Retention Analysis by Categories of Total Dairy Intake throughout the 4 Years Surrounding Pregnancy[1].**

| | Total Dairy Intake | | |
|---|---|---|---|
| | 1 s/m | 1 s/m -< 2 s/w | ≥ 2 s/w |
| N of live births | 3572 | 6189 | 8606 |
| | | Mean (SD) | |
| Age at birth (years) | 35.6 (3.9) | 35.2 (3.8) | 34.9 (3.8) |
| Pre-pregnant BMI ($kg/m^2$) | 23.2 (4.0) | 23.2 (3.8) | 23.1 (3.8) |
| Activity (h/w) | 2.3 (2.7) | 2.4 (2.6) | 2.5 (2.7) |
| AHEI score | 48.7 (10.9) | 48.4 (10.5) | 46.1 (10.0) |
| Energy intake (kcals/d) | 1583 (488) | 1809 (476) | 2115 (490) |
| Energy-adj protein (g/d) | 79.9 (14.5) | 83 (12.8) | 88.3 (11.9) |
| Energy-adj carbohydrate (g/d) | 233.5 (36.3) | 232.3 (31.7) | 232.2 (28.9) |
| Energy-adj total fat (g/d) | 62.3 (11.7) | 61.5 (10.8) | 59.3 (10.5) |
| Energy-adj trans-fat (g/d) | 3.3 (1.2) | 3.1 (1.1) | 2.9 (0.9) |
| Energy-adj alcohol (g/d) | 2.8 (5.5) | 2.9 (5.2) | 2.5 (5.0) |
| Total dairy (s/d) | 0.6 (0.3) | 1.4 (0.3) | 3.2 (0.9) |
| Yogurt (s/w) | 0.5 (0.8) | 1.1 (1.4) | 1.6 (2.1) |
| Milk (s/d) | 0.2 (0.2) | 0.8 (0.4) | 2.3 (0.9) |
| Cheese (s/d) | 0.3 (0.2) | 0.5 (0.3) | 0.7 (0.6) |
| Red and processed meat (s/d) | 0.9 (0.6) | 0.9 (0.6) | 1.0 (0.6) |
| Fruits and vegetables (s/d) | 3.8 (2.2) | 4.3 (2.2) | 4.6 (2.3) |
| SSB (bottles/d) | 1.2 (1.3) | 1.2 (1.2) | 1.5 (1.3) |
| Pre-pregnancy smoker (%) | 10.4 | 8.5 | 6.2 |
| First birth (%) | 29.4 | 28.1 | 23.8 |

Abbreviations: AHEI, alternative healthy eating index 2010; BMI, body mass index; Energy-adj, energy adjusted; SD, standard deviation; SSB, sugar sweetened beverages; s/d, servings per day; s/m, servings per month; s/w, servings per week.

[1] All the dietary variables and activities variables are cross-sectional; only BMI and smoking status are pre-pregnant.
